# Increasing the rate of datasets amenable to CT_FFR_ and quantitative plaque analysis: Value of software for reducing stair-step artifacts demonstrated in photon-counting detector CT

**DOI:** 10.1016/j.ejro.2024.100574

**Published:** 2024-06-04

**Authors:** Costanza Lisi, Lukas J. Moser, Victor Mergen, Thomas Flohr, Matthias Eberhard, Hatem Alkadhi

**Affiliations:** a Diagnostic and Interventional Radiology, University Hospital Zurich, University of Zurich, Zurich, Switzerland; b Department of Biomedical Sciences, Humanitas University, via Rita Levi Montalcini 4, 20090 Pieve Emanuele, Milan, Italy

**Keywords:** Coronary artery disease, computed tomography, coronary plaque, fractional flow reserve, quantitative analysis

## Abstract

**Purpose:**

To determine the value of an algorithm for reducing stair-step artifacts for advanced coronary analyses in sequential mode coronary CT angiography (CCTA).

**Methods:**

Forty patients undergoing sequential mode photon-counting detector CCTA with at least one stair-step artifact were included. Twenty patients (14 males; mean age 57±17years) with 45 segments showing stair-step artifacts and without atherosclerosis were included for CT_FFR_ analysis. Twenty patients (20 males; mean age 74±13years) with 22 segments showing stair-step artifacts crossing an atherosclerotic plaque were included for quantitative plaque analysis. Artifacts were graded, and CT_FFR_ and quantitative coronary plaque analyses were performed in standard reconstructions and in those reconstructed with a software (entitled *ZeeFree*) for artifact reduction.

**Results:**

Stair-step artifacts were significantly reduced in *ZeeFree* compared to standard reconstructions (p<0.05). In standard reconstructions, CT_FFR_ was not feasible in 3/45 (7 %) segments but was feasible in all *ZeeFree* reconstructions. In 9/45 (20 %) segments without atherosclerosis, the *ZeeFree* algorithm led to a change of CT_FFR_ values from pathologic in standard to physiologic values in *ZeeFree* reconstructions. In one segment (1/22, 5 %), quantitative plaque analysis was not feasible in standard but only in *ZeeFree* reconstruction. The mean overall plaque volume (111±60 mm^3^), the calcific (77±47 mm^3^), fibrotic (31±28 mm^3^), and lipidic (4±3 mm^3^) plaque components were higher in standard than in *ZeeFree* reconstructions (overall 75±50 mm^3^, p<0.001; calcific 51±42 mm^3^, p<0.001; fibrotic 22±19 mm^3^, p<0.05; lipidic 3±3 mm^3^, p=0.055).

**Conclusion:**

Despite the lack of reference standard modalities for CT_FFR_ and coronary plaque analysis, initial evidence indicates that an algorithm for reducing stair-step artifacts in sequential mode CCTA increases the rate and quality of datasets amenable to advanced coronary artery analysis, hereby potentially improving patient management.

## Highlights

• Advanced coronary analysis (CT_FFR_ & plaque analysis) requires high quality images.
• Image quality in sequentially acquired CCTA may be limited by stair-step artefacts.
• An algorithm reducing stair-step artifacts improves advanced coronary analysis.

## 1 Introduction

The majority of coronary computed-tomography angiography (CCTA) examinations are performed in the sequential (or step-and-shoot) mode [1], [2], which is characterized by a good trade-off between image quality and radiation dose [3]. However, in CT systems with detector width not covering the entire heart, image quality may be limited by stair-step artefacts. The prevalence of such artifacts in sequential mode CCTA depends on the temporal resolution and detector width of the respective scanner and is reported to occur in up to 18 % of patients [4] and 77 % of coronary segments, including different CT scanners and scanner generations [5].

Recently, an algorithm was introduced for reducing the extent and prevalence of stair-step artifacts (*ZeeFree*, Siemens), which is based on a non-rigid registration at the boundary of two adjacent image stacks [6]. Early experience with this algorithm indicated that the rate of stair-step artifact occurrence was significantly lower using the algorithm as compared to standard reconstructions [6]. However, that study did not include atherosclerotic plaques in the analysis, and did not test the impact of the algorithm on quantitative coronary plaque analysis and refined vessel evaluation such as computation of CT-based fractional flow reserve (CT_FFR_).

Non-invasive plaque characterization and quantification with CCTA has been demonstrated to be an important predictor for adverse coronary events in patients with ischemic cardiomyopathy [7], [8]. CT_FFR_ has been shown to improve the identification of hemodynamically significant stenoses compared to a pure morphological analysis alone [9], leading to a change in patient management in regard to revascularization [10]. Recent research focused on the development of automated image analysis tools for both CT_FFR_ and advanced coronary plaque analysis [11], [12], [13], [14]. However, relatively high failure rates were reported for CT_FFR_ analysis, ranging from 2.9 % [15] to 33 % [16], [17], mostly attributed to technical and image quality issues. Motion artifacts, heart rate variability, and higher slice thickness have been shown to be associated with non-feasible CT_FFR_ analyses [15], [17]. In regard to advanced coronary plaque analysis, a failure rate of around 5 % was reported, for similar reasons [18], [19]. These observations underline the importance of high CCTA image quality both to enhance the rate of datasets amenable to advanced analyses, and to improve the robustness and accuracy of quantitative results. Advanced coronary analysis has the potential to affect patient prognosis and survival through change of therapy leading to more appropriate invasive treatment or medication. Optimal therapeutic decisions are pivotal to achieve the best patient outcome in ischemic heart disease, as previously demonstrated [20], [21].

The purpose of our study was to determine the value of the new algorithm for reducing the prevalence and extent of stair-step artifacts in sequential mode CCTA for CT_FFR_ and quantitative coronary plaque analyses. We hypothesized that use of the algorithm will increase the rate of CCTA datasets amenable to these advanced coronary analyses with potentially relevant changes in quantitative results.

## 2 Materials and methods

### 2.1 Patient population

This retrospective study was performed at a tertiary academic hospital, had institutional review board and ethics committee agreement, and was conducted according to the Declaration of Helsinki principles. All patients provided written general informed consent for further use of their data for anonymized research. Two different patient populations were considered for study inclusion, and part of the patients were included also in a previous study on a different subject [6].

For CT_FFR_ analysis, patients who underwent sequential mode CCTA between August and October 2023 were screened for the presence of at least one coronary segment with a stair-step artifact in the absence of atherosclerotic disease and 20 patients were included (14 males; 6 females; mean age, 57 ± 17 years). In 14 patients the indication for CCTA was suspected coronary artery disease, while in the remaining six CCTA was performed for planning transcatheter aortic valve replacement.

For advanced coronary plaque analysis, patients who underwent sequential mode CCTA between February and October 2023 were screened for the presence of at least one coronary segment with a stair-step artifact crossing an atherosclerotic plaque and 20 patients were included (20 males; mean age, 74 ± 13 years). All patients were referred to CCTA for planning transcatheter aortic valve replacement. Patient demographics are presented in Table 1.

**Table 1:** Patient demographics **Plaque analysis CT**_**FFR**_.

**Characteristic**	**n=20**	**n=20**
Sex		
Female	0	6 (30 %)
Male	20 (100 %)	14 (70 %)
Age [years]	74 ± 13 (range 41–90)	57 ± 17 (range 30–86)
Body weight [kg]	79.1 ± 15.1 (range 60–109)	76.8 ± 12.8 (range 51–108)
Body mass index [kg/m^2^]	26.4 ± 4.3 (range 19.9–34.8)	25.9 ± 4.3 (range 18.5–38.9)
Heart rate during acquisition [bpm]	73.6 ± 16 (range 54–116)	73.9 ± 12.7 (range 55–97)
Medical history		
Arterial hypertension	19 (95 %)	0
Diabetes	7 (35 %)	8 (40 %)
Dyslipidemia	16 (80 %)	8 (40 %)
Smoking history	6 (30 %)	10 (50 %)

Note: Unless otherwise indicated, data are mean ± standard deviation or number of patients with percentages in parentheses. n = number of patients, bpm = beats per minute.

### 2.2 CT scan acquisition and image reconstruction

In the CT_FFR_ population, CCTA were acquired in the prospectively electrocardiography (ECG)-triggered ultra-high-resolution mode on a clinical dual-source photon-counting detector CT (NAEOTOM Alpha, Software Version VB10; Siemens Healthineers AG, Forchheim, Germany). Detector collimation was 120 ×0.2 mm. Tube voltage was 120kVp and automated tube current-modulation (CARE Dose4D, Siemens) was utilized with an image quality level of 64. Gantry rotation time was 0.25 seconds, achieving a temporal resolution of 66 ms. ECG pulsing was individually adapted to the heart rate. The median volume CT dose index (CTDI_vol_) was 31.2 mGy (interquartile range, 23.9–40.1 mGy). A triphasic contrast media protocol was used (60–100 mL iopromide, Ultravist 370 mg I/mL; Bayer Healthcare, Berlin, Germany) with injection rates from 3.2 mL/s to 6.0 mL/s, according to the patients’ body mass index. Acquisition was started with bolus tracking using a threshold of 140 Hounsfield units (HU) in the ascending aorta at 90kVp. All patients received sublingual nitroglycerin (2.5 mg isosorbide dinitrate) prior to the examination, unless contraindicated. No beta-blockers were administered. Reconstruction was done with a field of view (FOV) of 200 mm×200 mm, a matrix of 512 ×512 pixels, and a sharp vascular kernel (Bv60) with quantum iterative reconstruction at strength level 4. Slice thickness and increment were both 0.2 mm. Among the reconstructed phases, the single best phase showing least motion artifacts was selected and was reconstructed in the standard mode and applying the *ZeeFree* reconstruction algorithm (see details below).

In the advanced coronary plaque analysis population, scans were acquired in the prospectively ECG-triggered sequential mode either with UHR mode, as detailed above, or with standard resolution. Using the standard resolution mode, detector collimation was set to 144 mm×0.4 mm. All other acquisition parameters as well as the contrast media protocol were identical as described above. For standardization reasons, all scans in the plaque analysis population were reconstructed with a slice thickness 0.6 mm and an increment of 0.3 mm. A medium-sharp vascular kernel (Bv40) with QIR 3 was used with a FOV of 200 ×200 mm².

Presence and extent of stair step artifacts were graded in all patients by one reader (resident with four years of experience in cardiovascular imaging) using a 4-point visual grading scale, as previously shown [6]: a score of 1 indicated no stair-step artifacts, a score of 2 indicated small stair-step artifacts of less than 25 % of the vessel diameter, a score of 3 indicated moderate stair-step artifacts of less than the vessel diameter, and a score of 4 denoted a stair-step artifact with considerable discontinuity of the vessel.

### 2.3 The ZeeFree algorithm

Details of the technical background of the algorithm can be found elsewhere [22]. In principle, the entire heart is covered with ECG-triggered sequential scans. In successive cardiac cycles, image stacks with a width in the patient's longitudinal direction (z-direction) corresponding to the detector width are acquired that overlap by approximately 10 %. The *ZeeFree* correction algorithm applications consists of three steps. First, an image stack with the maximum width in the z-direction is reconstructed from the data of each cardiac cycle. In the overlapping area of the image stacks two images from consecutive cardiac cycles are available at each z-position [23]. Second, a displacement 3D vector field is obtained for each transition between two image stacks. At each stack-to-stack transition, a z-position at the center of the overlap region is defined. The difference between the two images from consecutive cardiac cycles at this z-position is minimized by a demon type registration algorithm [24]. Finally, all individually derived 3D vector fields are resampled to a single displacement 3D vector field for the whole heart, which is applied during image reconstruction according to the user defined parameters.

### 2.4 Advanced coronary data post-processing

Advanced plaque analysis was performed using a semiautomatic software (CT Coronary Plaque Analysis, software version 5.0.3, Siemens). The proximal and distal aspect of the atherosclerotic plaque was manually defined by a member of the study team (resident with four years of experience in cardiovascular imaging) in both reconstructions. The software then automatically detected the outer and inner vessel wall including the atherosclerotic plaque. Manual correction was performed if required. Plaque components were defined based on their attenuation value according to preset ranges: Non-calcific plaque components were considered as low-attenuating; lipid-rich when attenuation ranged between −100 HU and 29 HU, and fibrotic when attenuation ranged between 30 HU and 189 HU, as previously proposed [25]. Calcific plaques were defined at an attenuation above 190 HU. Plaque analysis output included the total plaque volume (mm^3^), calcific, non-calcific, lipidic and fibrotic plaque volume (mm^3^) and ratio (%).

For CT_FFR_ analysis, a prototype on-site machine learning-based algorithm (CT cFFR, software version 3.5; Siemens) was used [17]. The system automatically generates centerlines and luminal contours which can be edited by the reader, if considered necessary. All coronary stenoses have to be marked. CT_FFR_ values are computed at all locations in the coronary tree and the resulting values are color-coded in an anatomical model. One reader (resident with four years of experience in cardiovascular imaging) assessed CT_FFR_ for all included patients in both reconstructions. Two coronary trees (standard and *ZeeFree* mode) were created for each patient and a cut-off value of 0.80 was applied to distinguish between positive and negative segment-specific CT_FFR_ [26].

### 2.5 Statistical analysis

Quantitative variables are expressed as mean ± standard deviation or median and interquartile range (IQR), as applicable. Qualitative variables are reported as counts or percentages. Wilcoxon tests were used to compare ordinal and continuous variables. Paired samples t test served to compare means. Statistical significance was assumed at a two-tailed P-value below 0.05. All statistical analyses were performed using commercially available software (IBM SPSS Statistics, version 29.0.2.0).

## 3 Results

### 3.1 *CT*_*FFR*_*analysis*

**Fig. 1:**
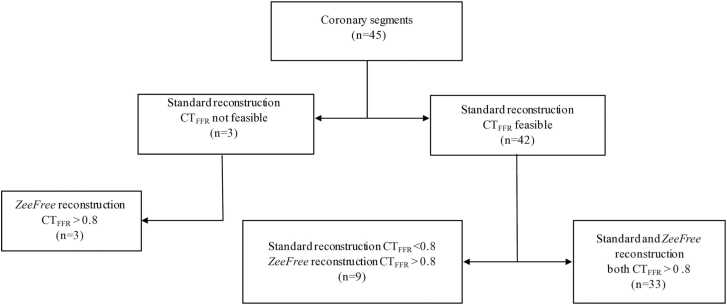
Flow chart of the CT_FFR_ study part.

**Fig. 2:**
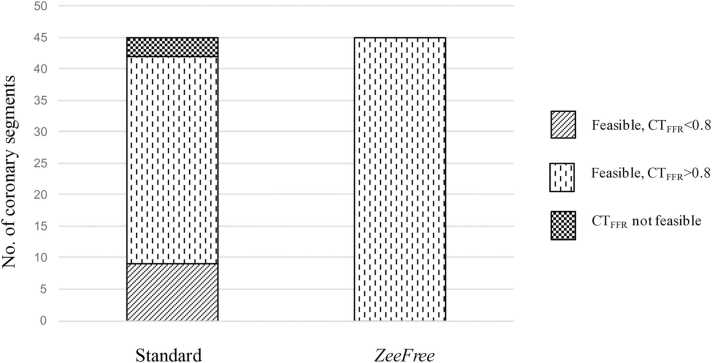
Number of coronary segments with stair step artifacts being feasible or not feasible for CT_FFR_ analyses. Note the lack of coronary segments being not feasible for CT_FFR_ analyses and the relevant change of normal vs. pathological CT_FFR_ values in the *ZeeFree* reconstructions.

Twenty patients and 45 coronary segments showing stair-step artifacts were included, and CT_*FFR*_ analysis results are shown in Table 2. Stair step artifact scores were graded significantly lower for all segments from standard to *ZeeFree* reconstructions except for one case (median score 3 for standard and score 1 for *ZeeFree* reconstructions, p<0.05). CT_FFR_ analysis results are illustrated in Fig. 1. In standard reconstructions, CT_FFR_ analysis was not feasible in 3/45 (7 %) segments while with *ZeeFree* reconstructions CT_FFR_ was feasible in all 45/45 (100 %) segments. Among the none-feasible CT_FFR_ cases with standard reconstruction, all segments had physiological CT_FFR_ values (>0.80) in *ZeeFree* reconstructions. In 33/45 (73 %) of segments, both standard and *ZeeFree* reconstructions yielded similar CT_FFR_ results (>0.80). In 9/45 (20 %) segments, the *ZeeFree* algorithm led to a change of CT_FFR_ values from pathologic (<0.80) in standard to physiologic (>0.80) with *ZeeFree* (Fig. 2).

**Table 2:** CT_FFR_ analysis results in both reconstructions.

				**Standard**			***ZeeFree***	
**Patient number (n=20)**	**Segment number (n=45)**	**Artery and AHA segment with artifact**	Artifact grade*	**CT**_**FFR**_ **feasibility**	**CT**_**FFR**_**value**	Artifact grade*	**CT**_**FFR**_ **feasibility**	**CT**_**FFR**_**value**
**1**	1	RCA 1	3	yes	0.99	1	yes	0.99
	2	LAD 8	3	yes	0.95	1	yes	0.94
	3	CX 13	2	yes	0.99	2	yes	0.97
**2**	4	LAD 8	2	yes	0.93	1	yes	0.92
	5	CX 13	2	yes	0.97	1	yes	0.97
**3**	6	RCA 2	3	yes	0.99	1	yes	0.99
	7	RCA 3	2	yes	0.99	1	yes	0.98
**4**	8	RCA 3	3	yes	0.98	1	yes	0.98
	9	LAD 8	2	yes	0.92	1	yes	0.92
	10	CX 12	2	yes	0.95	1	yes	0.97
**5**	11	RCA 2	3	yes	0.98	2	yes	0.98
	12	RCA 3	3	yes	0.96	2	yes	0.96
	13	LAD 7	2	yes	0.97	1	yes	0.97
	14	LAD 8	3	yes	0.94	2	yes	0.96
**6**	15	RCA 1	2	yes	0.99	1	yes	0.99
	16	LAD 6	3	yes	0.42	2	yes	0.98
	17	LAD 7	2	yes	0.15	1	yes	0.82
	18	CX 11	3	yes	1	2	yes	0.99
**7**	19	RCA 4	2	yes	0.83	1	yes	0.82
**8**	20	RCA 2	3	yes	0.99	2	yes	0.99
	21	CX 13	2	yes	0.97	2	yes	0.95
**9**	22	LAD 8	2	yes	0.94	1	yes	0.95
**10**	23	RCA 2	3	yes	0.98	1	yes	0.99
	24	RCA 3	3	yes	0.98	1	yes	0.98
	25	LAD 8	2	yes	0.92	1	yes	0.94
**11**	26	CX 12	2	yes	0.98	1	yes	0.99
**12**	27	RCA 3	2	yes	0.97	1	yes	0.99
	28	LAD 8	4	NF	NF	4	yes	0.4
**13**	29	RCA 1	2	yes	1	1	yes	0.99
	30	LAD 7	3	yes	0.75	1	yes	0.95
	31	LAD 8	2	yes	0.56	1	yes	0.91
	32	CX 11	4	yes	0.91	1	yes	0.99
**14**	33	RCA 2	2	yes	0.96	1	yes	0.98
**15**	34	LAD 7	4	yes	0.94	1	yes	0.98
**16**	35	RCA 2	2	yes	0.99	1	yes	0.97
**17**	36	RCA 2	2	yes	0.99	1	yes	0.99
**18**	37	LAD 7	4	yes	0.77	1	yes	0.80
	38	LAD 8	4	yes	0.79	1	yes	0.80
	39	CX 12	4	NF	NF	1	yes	0.92
	40	CX 13	4	NF	NF	1	yes	0.87
**19**	41	RCA 2	3	Yes	0.98	1	yes	0.96
	42	LAD 6	4	Yes	0.52	2	yes	0.99
	43	LAD 7	4	Yes	0.46	1	yes	0.85
	44	CX 12	3	Yes	0.43	1	yes	0.98
**20**	45	RCA 1	2	Yes	0.98	1	yes	0.99

CTFFR=computed tomography fractional flow reserve; NF=not feasible;

* stair-step artifacts were graded from 1 to 4 (1=minimal, 2=mild, 3=moderate, 4=severe).

**Fig. 3:**
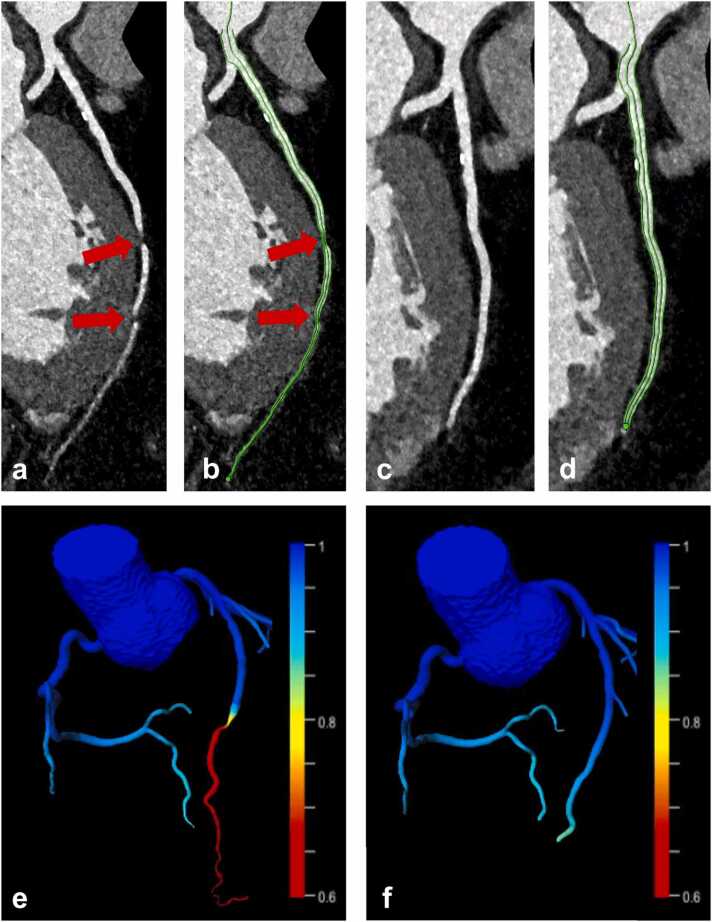
64-year-old male patient with intermediate risk profile and exertional dyspnea undergoing sequential mode CCTA. Two severe (grade 4) stair-step artifacts were present in standard reconstructions in the distal LAD (**a**, arrows), which were resolved with the *ZeeFree* reconstructions (**c**). Vessel centerline and lumen contouring for CT_FFR_ analyses suggesting two severe narrowings of the LAD at the artifact levels in standard mode reconstructions (**b**, arrows), while these artifacts and wrong segmentations are no longer present in *ZeeFree* reconstructions (**d**). CT_FFR_ analysis indicates pathological flow in standard reconstructions with critical values <0.8 at the levels of the artifacts (**e**), whereas *ZeeFree* reconstructions indicate normal flow (>0.80) (**f**).

**Fig. 4:**
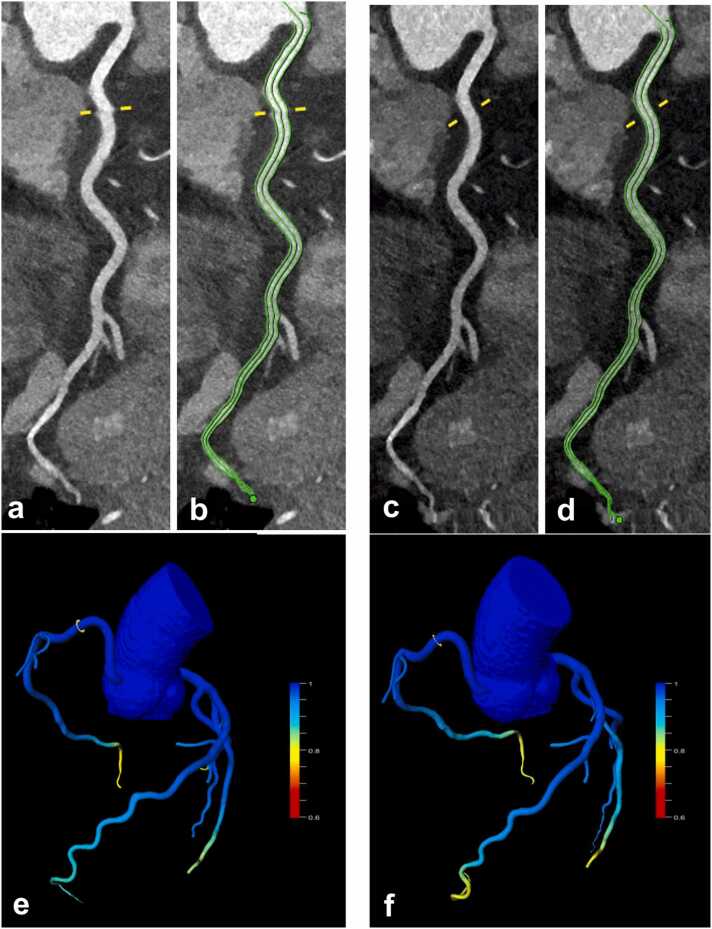
45-year-old male patient with episodes of atypical chest pain during physical exertion undergoing sequential mode CCTA. Small (grade 2) stair step artifacts can be seen in the proximal RCA in standard mode reconstructions (**a**, yellow line), which is resolved with the *ZeeFree* algorithm (**c**). Vessel center-line and lumen contouring for CT_FFR_ analyses (**b,d**). CTFFR indicated similarly physiologic values (0.99) at the level of the stair-step artifact in both reconstructions (**e,f**).

Fig. 3 illustrates a case with a falsely pathologic CT_FFR_ value induced by artificial stenosis from severe stair step artifacts, which could be corrected with the *ZeeFree* algorithm. Fig. 4 illustrates a case where both standard and *ZeeFree* reconstructions yielded normal CT_FFR_ values despite of small stair step artifacts in standard reconstructions.

### 3.2 Quantitative coronary plaque analysis

**Fig. 5:**
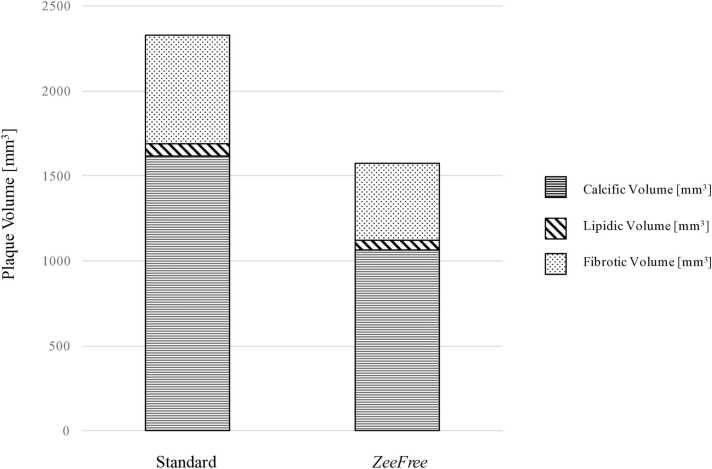
Calcific, lipidic, and fibrotic plaque component volumes in standard and *ZeeFree* reconstructions. Note the reduction in overall and in selective plaque components in the images without artifacts.

Twenty patients and 22 coronary segments containing plaques with stair-step artifacts were included in this analysis, and quantitative plaque analysis results are shown in Table 3. In one segment with a severe stair step artifact (grade 4), quantitative plaque analysis was not feasible in standard but only in *ZeeFree* reconstructions. Stair step artifact scores were significantly downgraded for all segments from standard to *ZeeFree* reconstructions (median score 2 for standard and score 1 for *ZeeFree* reconstructions, p<0.05). Mean overall plaque volume (111±60 mm^3^) and volumes of the calcific (77±47 mm^3^), non-calcific (34±30 mm^3^), and fibrotic plaque components (31±28 mm^3^) were significantly higher in standard as compared to those in *ZeeFree* reconstructions (overall plaque volume 75±50 mm^3^, p<0.001; calcific 51±42 mm^3^, p<0.001; non-calcific 24±21, p<0.01, fibrotic plaque component 22±19 mm^3^, p<0.05). A similar trend was found for the lipidic plaque component without reaching statistical significance (standard reconstruction: lipidic volume 4±3 mm^3^; *ZeeFree* reconstruction: lipidic volume 3±3 mm^3^, p=0.055) (Fig. 5).

**Table 3:** Quantitative plaque analysis results in both reconstructions.

			**Standard**						***ZeeFree***					
**Patient (n=20)**	**Segment (n=22)**	**Artery and AHA segment with artifact**	Artifact grade*	**Total plaque volume [mm**^**3**^**]**	**Calcific component [mm**^**3**^**]**	**Non-calcified component [mm**^**3**^**]**	**Lipidic component [mm**^**3**^**]**	**Fibrous component [mm**^**3**^**]**	Artifact grade*	**Total plaque volume [mm**^**3**^**]**	**Calcified component [mm**^**3**^**]**	**Non-calcified component [mm**^**3**^**]**	**Lipidic component [mm**^**3**^**]**	**Fibrous component [mm**^**3**^**]**
**1**	1	RCA 2	2	230.82	183.52	47.3	5.63	41.67	1	96.71	89.32	7.39	0.38	7.01
**2**	2	LAD 6	2	51.3	29.9	21.4	2.13	19.27	1	35.58	11.05	24.53	2.17	22.36
**3**	3	CX 11	2	105.30	95.04	10.26	0.64	9.62	1	54.14	48.06	6.08	0.85	5.23
**4**	4	LAD 8	2	35.68	21.45	14.23	1.65	12.50	1	18.92	7.05	11.87	0.76	11.11
**5**	5	RCA 2	2	55.38	40.72	14.66	1.46	13.22	1	26.83	14.76	12.07	0.14	11.93
**6**	6	RCA 2	3	145.06	85.32	59.74	12.12	47.62	1	102.45	60.88	41.57	9.32	32.25
**7**	7	LAD 7	2	106.32	95.45	10.87	1.57	9.38	1	68.72	55.67	13.05	0.87	12.18
	8	CX 11	2	63.36	58.35	5.01	0.73	4.28	1	37.73	22.92	14.81	1.50	13.31
**8**	9	LAD 7	2	92.7	79.3	18.8	5.11	13.69	1	73.14	54.34	18.8	1.95	16.85
**9**	10	LAD 6	2	87.42	69.25	18.17	3.76	14.41	1	62.67	48.10	14.57	3.55	11.02
**10**	11	CX 11	3	96.35	85.86	10.49	0	10.49	1	51.94	50.03	1.91	0.38	1.91
**11**	12	LAD 6	2	126.9	87.42	39.48	2.37	37.11	1	79.65	52.83	26.82	5.41	21.41
**12**	13	CX 12	4	NF	NF	NF	NF	NF	2	32.63	22.52	10.11	1.11	9,0
**13**	14	RCA 1	3	114.44	83.12	31.32	1.94	29.38	2	80.79	64.51	16.28	1.08	15.20
**14**	15	RCA 1	3	60.10	34.75	25.35	1.88	23.47	2	38.90	23,0	15.90	0.98	14.92
**15**	16	RCA 1	2	104.47	66.23	38.24	3.72	34.52	1	75.09	46.55	28.54	3.63	24.91
**16**	17	LAD 6	3	66.21	54.79	11.31	1.02	10.29	1	39.71	27.03	12.68	1.16	11.52
**17**	18	RCA 2	2	31.17	17.95	13.22	0.33	12.89	1	17.31	3.02	14.29	0.66	13.63
**18**	19	LAD 6	4	240.22	203.1	37.12	5.43	31.69	1	222.8	198.34	24.53	4.4	20.13
	20	RCA 2	3	193.24	119.95	73.29	5.39	67.9	1	164.17	105.17	59.4	3.38	56.02
**19**	21	RCA 3	3	155.57	38.92	116.65	11.41	105.24	1	124.90	45.16	79.74	3.34	76.40
**20**	22	LAD 6	4	163.33	64.33	99	8.16	90.84	1	101.33	36.38	64.95	8.82	56.13

NF: not feasible.

* Stair-step artifacts were graded from 1 to 4 (1=minimal, 2=mild, 3=moderate, 4=severe).

**Fig. 6:**
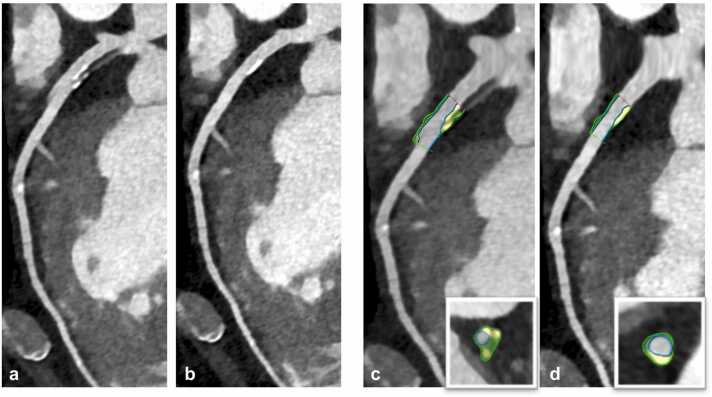
76-year-old male patient with severe aortic stenosis planned for transcatheter aortic valve replacement. Pre-procedural sequential mode CCTA showed a mixed plaque in the proximal LAD with a severe stair-step artifact in standard mode reconstructions (**a**), eliminated with the *ZeeFree* algorithm (**b**). Semiautomatic quantitative plaque analysis in standard and *ZeeFree* reconstructions is shown (**c,d**) in curved and short-axis reformations (inserts in **c,d**). As can be appreciated, both volume and plaque composition changes in the images without artifacts (standard total plaque volume=163.33 mm^3^; *ZeeFree* total plaque volume=101.33 mm^3^).

**Fig. 7:**
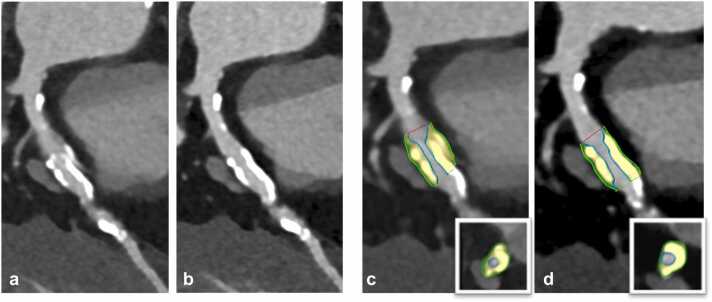
78-year-old male patient with aortic stenosis planned for transcatheter aortic valve replacement. Pre-procedural sequential mode CCTA is performed showing a severe stair step artifact through a calcific plaque in the proximal LAD in standard mode reconstructions (**a**), which is eliminated with the *ZeeFree* algorithm (**b**). Semiautomatic quantitative plaque analysis in standard and *ZeeFree* reconstructions are shown (**c,d**) in both curved and short-axis reformations (inserts in **c,d**). Note the change in size and shape of the calcified plaque in *ZeeFree* reconstructions (standard total plaque volume=240.22 mm^3^; *ZeeFree* total plaque volume=222.8 mm^3^).

Two representative examples of coronary plaques with stair step artifacts in standard reconstructions which could be resolved in *ZeeFree* reconstructions, along with changes in quantitative plaque volumetry, are illustrated in Fig. 6, Fig. 7.

## 4 Discussion

Accurate advanced coronary artery and coronary plaque analysis can predict adverse coronary events in patients with ischemic cardiomyopathy, improve the identification of hemodynamically significant stenosis compared to a pure morphological analysis alone, and may change patient management in regard to revascularization [7], [8], [9], [10]. Increased advanced plaque analysis feasibility and accuracy may lead to improved therapy for patients with stable chest pain [7], and improved CT_FFR_ feasibility could facilitate its integration in the clinical workflow hereby improving the identification of hemodynamically significant stenosis [9]. It is known that accurate advanced coronary artery and coronary plaque analysis requires high quality datasets without artifacts, and many factors such as patient heart rate, timing of contrast media administration, temporal and spatial resolution of the CT scanner, and reconstruction techniques have shown to affect advanced coronary CT_FFR_ and plaque analysis feasibility [15], [16], [19]. Given the current trend in developing artificial intelligence-based, automatic post-processing techniques for such advanced analyses [27], high-quality CCTA images are also relevant to increase the feasibility rate for such image data post-processing [12], [17]. Our study indicates that the occurrence of a commonly encountered problem in sequential mode CCTA, i.e. stair-step artifacts, can be considerably reduced when using a novel algorithm for image processing, with improved results for both CT_FFR_ and plaque analysis.

In the CT_FFR_ study part, stair-step artifacts were significantly reduced in all but one case when using the new algorithm for image reconstruction. CT_FFR_ analysis was not feasible in 7 % of standard reconstruction cases because of such artifacts, but was feasible in 100 % of the *ZeeFree* reconstructions. Importantly, in 20 % of all cases, the *ZeeFree* algorithm led to a change of CT_FFR_ values from pathologic (<0.80) in standard to physiological (>0.8) with *ZeeFree*. Despite lacking the gold standard catheter-FFR in our patients, we assume that the physiological CT_FFR_ values in our patients were more likely correct, since the coronary arteries showed no significant stenosis and no atherosclerotic plaques in the segments with pathologic CT_FFR_ values in standard reconstructions. We believe that the CT_FFR_ algorithm calculated pathological CT_FFR_ values because the artifacts were considered high grade stenosis (which can be appreciated in Fig. 3). While in this study setting it would have been easy to recognize a false positive CT_FFR_ result, the *ZeeFree* algorithm has the potential to also reduce the number of false-positive CT_FFR_ cases in patients with stair-step artifacts adjacent to coronary artery stenosis, in which a false positive CT_FFR_ value may not be easily perceptible.

In the quantitative coronary plaque analysis part, post-processing was not feasible in one segment because of a severe stair-step artifact that was resolved in *ZeeFree* reconstructions. Interestingly, the overall plaque volume as well as the volumes of each individual plaque component (i.e., calcific, fibrotic, and lipidic) were significantly larger in standard than in *ZeeFree* reconstructions. Despite the lack of a reference standard such as optical coherence tomography (OCT) or intra-vascular ultrasound (IVUS) for comparison available, we think that the quantitative results in the *ZeeFree* images are more likely correct. Stair-step artifacts often induce a “doubling effect” of coronary segments and plaques over the artifact, leading to possible volume overestimation in standard reconstructions (which can be appreciated in Fig. 6, Fig. 7). One could argue that no reader would perform quantitative analyses on plaques containing obvious stair-step artifacts. However, using recently developed automatic quantitative plaque analysis software tools for CCTA will profit from images containing no such artifacts, hereby improving the accuracy of their results.

The following study limitations merit consideration. First, this is a single-center study, conducted on a limited sample size, which may limit the generalizability of our findings. Second, both the algorithm evaluated in this study and the CT scanner is limited to a single vendor. Also, the algorithm described herein is not unique to one CT scanner type but was designed also for other scanner generations such as energy-integrating detector CT. Third, CT_FFR_ and quantitative coronary plaque analysis were performed with a single vendor analysis tool, despite other software tools being available for these purposes as well. Fourth, no follow-up data of our population was available and thus, no improvement in outcome could be demonstrated. Furthermore, the results were derived from a selected patient population who was specifically screened for the presence of stair-step artifacts. Thus, the reported prevalence of such artifacts in our study is considerably biased, and rate of CCTA datasets profiting from the described algorithm in real clinical life is expected to be lower. Finally, the effect of the algorithm on the diagnostic accuracy relative to the reference standard for plaque analysis, such as OCT [28] or IVUS [29] was not evaluated, similarly to the lack of catheter-FFR [30] being not available for comparison with CT_FFR_.

## 5 Conclusion

In conclusion, our initial results indicate that a recently developed algorithm for reducing the prevalence and extent of stair-step artifacts in sequential mode CCTA improves the quality of images and increases the datasets amenable to advanced coronary plaque and coronary hemodynamic assessment, eventually leading to substantial changes in quantitative plaque and flow information. Future studies are needed to confirm our results in a larger population and to demonstrate the benefits of the algorithm also in other CT scanners. A comparison of CT_FFR_ and quantitative coronary plaque analysis results with their reference standard modalities (OCT, IVUS, catheter-FFR) are also needed to further confirm the accuracy of our results. Finally, prospectively designed studied may be useful to demonstrate its effect for patient outcomes.

## Funding statement

Costanza Lisi reports financial support was provided by European School of Radiology-BRACCO Research Fellowship 2024. Diagnostic and Interventional Radiology Department of University Hospital Zurich reports a relationship with Bayer, Canon, Guerbet, Siemens that includes: funding grants. Hatem Alkadhi and Matthias Eberhard report a relationship with Siemens Healthineers AG that includes: speaking Honorarium.

## Ethical statement

The work described has been carried out in accordance with The Code of Ethics of the World Medical Association (Declaration of Helsinki) for experiments involving humans. This retrospective study had institutional review board and ethics committee agreement.All patients provided general consent for further use of their data for anonymized research.

## CRediT authorship contribution statement

**Costanza Lisi:** Writing – review & editing, Writing – original draft, Visualization, Methodology, Investigation, Formal analysis, Conceptualization. **Lukas Jacob Moser:** Writing – review & editing, Methodology. **Hatem Alkadhi:** Writing – review & editing, Writing – original draft, Supervision, Methodology, Conceptualization. **Matthias Eberhard:** Writing – review & editing, Supervision. **Thomas Flohr:** Writing – review & editing. **Victor Mergen:** Writing – review & editing.

## Declaration of Competing Interest

The authors declare the following financial interests/personal relationships which may be considered as potential competing interests: Diagnostic and Interventional Radiology Department of University Hospital Zurich reports a relationship with Bayer, Canon, Guerbet, Siemens that includes: funding grants. Hatem Alkadhi and Matthias Eberhard report a relationship with Siemens Healthineers AG that includes: speaking Honorarium.
